# Health system wide “big data” analysis of rheumatologic conditions and scleritis

**DOI:** 10.1186/s12886-020-01769-3

**Published:** 2021-01-06

**Authors:** Meghan K. Berkenstock, Andrew R. Carey

**Affiliations:** 1 Division of Ocular Immunology, Wilmer Eye Institute, 600 N. Wolfe St., Maumenee Building, 3rd floor, Baltimore, MD 21287 USA; 2 Division of Neuro-Ophthalmology, Wilmer Eye Institute, Baltimore, MD USA

**Keywords:** Autoimmune disease, Scleritis, Disease- associations

## Abstract

**Background:**

The development of scleritis in the setting of autoimmune conditions has been well documented. Prior series have assessed the relationship between systemic autoimmune disorders and scleritis only in patients referred for rheumatologic or ocular inflammation. This can lead to a referral bias. We reviewed all charts within the electronic medical record (EMR) of a health system for patients with systemic autoimmune and scleritis diagnoses to determine the prevalence of both and which disorders had the highest relative risk of developing scleritis.

**Methods:**

The EMR was searched for scleritis and systemic inflammatory diagnoses in the past medical history and diagnosis tabs, and for associated disease specific laboratory values. The intersection of scleritis and systemic inflammatory conditions was assessed through searching both SNOMED Clinical Terminology and ICD-10 codes for diagnoses. The prevalence of each autoimmune disorder, scleritis prevalence, the percentage of patients with an autoimmune condition having scleritis, the percentage of patients with scleritis having an autoimmune condition; the relative risk (RR) of scleritis patients having a specific autoimmune disorder were calculated.

**Results:**

A total of 5.9 million charts were searched with autoimmune conditions identified in 148,993 patients. The most common autoimmune conditions overall were HLA-B27-associated diseases (*n* = 26,680; prevalence 0.45%); rheumatoid arthritis (RA)(*N* = 19,923; prevalence 0.34%). Conversely, 2702 patients were identified with scleritis (prevalence 0.05%), of which 31.4% had an associated autoimmune condition. Patients with RA represented the highest percentage of patients with an autoimmune condition having scleritis. Granulomatosis with polyangiitis (GPA) represented the highest the percentage of patients with scleritis having an autoimmune condition. Sjogrens was the third most common condition associated with scleritis- making up 4.5% of cases. An association with juvenile idiopathic arthritis (JIA) was seen in 0.3% of patients.

**Conclusions:**

While this is the largest retrospective review examining the association between autoimmune disease and scleritis, the findings are similar to prior studies with nearly a third of scleritis patients having an underlying autoimmune diagnosis. Limitations of the study included accurate chart coding; having laboratory results within the searchable EMR. Future research is needed to delineate associations of systemic disease with the anatomic location of scleritis using EMR.

## Background

The development of scleritis in the setting of autoimmune conditions has been well documented [1–10]. An underlying diagnosis associated with the development of scleritis has been identified between 29 and 57% of patients [3, 5–7, 9–13]. The most common rheumatologic diseases associated with scleritis vary by the study population, location, practice setting, and the years of patient follow-up [7, 9, 11, 12, 14, 15]. However, in the largest cohort to date, the most commonly diagnosed rheumatologic conditions were rheumatoid arthritis (RA), systemic lupus erythematous (SLE), and granulomatosis with polyangiitis (GPA) [12]. Importantly, these diagnoses were also associated with a high rate of not achieving scleritis remission [12].

Many of these series have assessed the relationship between systemic autoimmune disorders and scleritis in patients referred for rheumatologic evaluation and to rule out ocular inflammation [2–16]. Limiting studies to only these two populations leads to introduction of a referral bias [7]. A study assessing the prevalence and the association of systemic autoimmune disease with the development of scleritis by examining the records of all patients seen at tertiary referral center has not been reported.

Thus, we reviewed all of the electronic medical records within the Johns Hopkins Health System to extract patients with both systemic, autoimmune, and scleritis diagnoses. From these, we determined the prevalence of both the autoimmune disorders in addition to scleritis. We further examined which of these systemic disorders had the highest relative risk of developing scleritis.

## Methods

The electronic medical record (EMR) at Johns Hopkins Medicine Health System was queried using the SlicerDicer application which allowed a search of de-identified medical records from 1/1/2013, when the hospital switched to an EMR, through 8/16/2018. Medical records were searched for scleritis; systemic inflammatory conditions in the past medical history or diagnosis tabs, and associated specific laboratory values;  and the intersection of scleritis and systemic inflammatory conditions. Diagnoses were searched by both SNOMED Clinical Terminology (an alternate diagnostic coding system which facilitates interoperability of the EMR) and ICD-10 codes. The query terms are shown in Table 1. Systemic lupus and subacute lupus were grouped together. The HLA-B27- associated diseases were all grouped together under HLA-B27. Scleroderma, systemic sclerosis, and CREST were grouped together under scleroderma. PMR and GCA were grouped together under PMR/GCA. The Sjogren diagnoses (Sicca with myopathy, Unspecificed sicca, Sicca with ketatoconjunctivitis, and Sjogren with organ involvement) were grouped together. Keratitis was not included due to concern of inconsistent coding that may have included infectious causesor inflammatory causes not included in this study, non-inflammatory dry eye, and allergic causes.

**Table 1:** Ocular and systemic diagnostic search terms and ICD-10 codes

Ocular diagnostic codes	Systemic search terms	Labs search terms
Scleritis (SNOMED)	Rheumatoid arthritis / M05, M06	RF, CCP
Anterior scleritis H15.01	^a^ Systemic lupus erythematosus / M32	dsDNA, Smith, RNP
Posterior Scleritis H15.03	^a^ Subacute cutaneous lupus / L93	dsDNA, Smith, RNP
Other Scleritis H15.09	^b^ Ankylosing spondylitis / M45	HLA-B27
Scleritis with corneal involvement H15.04	^b^ Psoriatic arthritis / L40.5	HLA-B27
	^b^ Ulcerative colitis / K51	HLA-B27
	^b^ Crohn disease / K50	HLA-B27
	^b^ Inflammatory bowel disease	HLA-B27
	^b^ Reactive arthritis / M02.3	HLA-B27
	Gout / M10	Uric Acid
	Juvenile rheumatoid arthritis (JRA) / Juvenile inflammatory arthritis (JIA) / M08; Juvenile polyarthritis M08.3; Pauciarticular JRA;	
	^c^ Polymyalgia rheumatica (PMR) / M35.3	
	^c^ Giant cell arteritis (GCA) / M31.5 & M31.6	
	Sjogren syndrome / M35.0; Unspecificied Sicca / M35.00; Sicca with Keratoconjunctivitis Sicca/ M35.01; Sicca with lung involvement / M35.02; Sicca with Myopathy/ M35.03; Sicca with renal involvement / M35.04; Sicca with other Organ Involvement/ M35.09	SS-A, SS-B, Ro, Ro52, Ro50, La
	Scleroderma, Systemic Sclerosis, CREST / L94.0, L94.1, L94.2, L94.3, M34	Scl-70, Centromere, RNA Polymerase III
	Sarcoidosis / D86	ACE, Lysozyme
	Granulomatosis with polyangiitis (GPA) / M31.3	C-ANCA, PR3
	Multiple sclerosis (MS) / G35	
	Behcet disease / M35.2	HLA-B51
	Polyarteritis nodosa (PAN) & Microscopic polyangiitis (MPA) / M30, M31.7	P-ANCA, MPO
	Mixed Connective Tissue Disorder / M35.1, M35.8	

^a^Grouped together under Lupus, ^b^Grouped together under HLA-B27, ^c^ Grouped together under PMR/GCA

After totaling the number of individual charts for each condition, the intersection of each systemic autoimmune condition and scleritis was then queried and tallied. The statistics calculated from the data included: the prevalence of systemic autoimmune conditions, the prevalence of scleritis, the percentage of patients with an autoimmune condition having scleritis, the percentage of patients with scleritis having an autoimmune condition, the relative risk (RR) of patients with scleritis also having a specific autoimmune condition with an associated 95% confidence interval (95% CI), and chi-square *p*-values. E-values were also calculated for the RR and lower limit of the 95% CI to estimate how strong a confounder would have to be to reduce the association to chance [17].

## Results

A total of 5.9 million charts were searched, and of these, 148,993 patients were identified with an autoimmune condition. The most common autoimmune condition was gout (*n* = 47,712, prevalence 0.81%), HLA-B27-associated diseases (*n* = 26,680; prevalence 0.45%) and RA (*N* = 19,923; prevalence 0.34%). The least common was Behcet disease (*n* = 317), PAN (*n* = 365), and GPA (*n* = 859). Conversely, 2702 patients were identified with scleritis (prevalence 0.05%), of which 30.8% had an associated autoimmune condition. The raw data for all diagnoses, number of scleritis cases, percentage of total scleritis associated with each systemic condition, and prevalence of scleritis within each condition are listed in Table 2. Data are listed in decreasing magnitude of overall scleritis cases.

**Table 2:** Percent of Patients with Scleritis and Systemic Condition

Condition	N	Scleritis cases	% of scleritis cases	% of disease with scleritis
Total	5,917,914	2702	31.4%	0.05%
RA	19,923	184	6.8%	0.9%
HLA-B27	26,680	153	5.7%	0.6%
Sjogren	10,576	121	4.5%	1.1%
Gout	47,712	95	3.5%	0.2%
Lupus	11,696	80	3.0%	0.7%
GPA	859	45	1.7%	5.2%
Sarcoid	7699	40	1.5%	0.5%
MS	9980	30	1.1%	0.3%
PMR/GCA	4456	29	1.1%	0.7%
MCTD	4061	27	1.0%	0.7%
Scleroderma	4232	17	0.6%	0.6%
PAN	365	11	0.4%	3.0%
Behcet	317	7	0.3%	2.2%
JIA/JRA	1351 (23 HLA-B27+)	9 (0 HLA-B27+)	0.3%	0.7%

*RA* Rheumatoid arthritis, *GPA* Granulomatosis with polyangiitis, *MS* Multiple sclerosis, *MCTD* Mixed connective tissue disorder, *PAN* Polyarteritis nodosa, *PMR/GCA* Polymyalgia rheumatica & giant cell arteritis, *JIA/JRA* Juvenile inflammatory arthritis / juvenile rheumatoid arthritis

The most frequent cause of scleritis was RA making up 6.8%, followed by HLA-B27 with 5.7% of cases, and Sjogren / Sicca with 4.5% of cases. The rarest causes were JIA and Behcet  disease at 0.3% of cases followed by PAN at 0.4%.

Many systemic conditions had high association with scleritis. GPA was highest, followed by PAN, Behcet Disease, Sjogren, and RA. RR ranged from 117–20 with all *p*-values < 0.1 × 10^− 10^. Full results for associations with 95% confidence intervals, *p*-values, e-values, and e-values for the 95% CI lower limit are available in Table 3.

**Table 3:** Relative risk of patient with scleritis having a specified underlying systemic condition

Condition	RR	95% CI	p	E-value for RR	E-value for 95% lower CI
GPA	115	86–153	0	229	172
PAN	66	36.8–118	3.00E-155	131	73
Behcet	48	23.2–101	1.00E-72	96	46
Sjogrens	25.1	20.9–30	0	50	41
RA	20.2	17.4–23.5	0	40	34
Lupus	15	12–18.7	2.00E-229	29	23
MCTD	14.6	10–21.2	3.00E-76	28	19
JIA/JRA	14.6	7.6–28	1.00E-26	29	15
PMR / GCA	14.3	9.9–20.5	8.60E-80	28	19
HLA-B27	12.6	107–14.8	0	25	21
Sarcoid	11.4	8.3–15.5	1.00E-84	22	16
Scleroderma	8.8	5.5–14.2	2.00E-27	17	10
MS	6.6	4.6–9.4	8.00E-33	13	9
Gout	4.4	3.6–5.3	6.00E-56	8	7

*RR* Relative risk, *CI* Confidence interval, *RA* Rheumatoid arthritis, *GPA* Granulomatosis with polyangiitis, *MS* Multiple sclerosis, *MCTD* Mixed connective tissue disorder, *PAN* Polyarteritis nodosa, *PMR/GCA* Polymyalgia rheumatica & giant cell arteritis, *JIA/JRA* Juvenile inflammatory arthritis / juvenile rheumatoid arthritis

## Conclusion

To our knowledge, this is the largest retrospective review examining the association between rheumatologic disease and scleritis. The previous literature is comprised of retrospective analyses of patients seen only within rheumatology or ocular immunology clinics, potentially leading to a referral bias [2–16]. In contrast, our study included all established patients within the Johns Hopkins Health System over a five-year period, which yielded a study population of 5.9 million patients. While our institution has a rheumatology department with subspecialized care centers and an ocular immunology division, the power of a larger patient database leads to a cohort that is more representative of the general medical population. It is not lost that our cohort is derived from a medical database, which is not representative of the general population. Thus, some but not all inherent referral bias cannot be removed from the analyses. Hence our higher incidence of scleritis compared with the estimated incidence of scleritis in the United States (0.05% versus 0.012%) [12].

The most common systemic condition associated with the development of scleritis in previous studies has been RA [5, 7, 9, 11–13, 15]. We found a very similar percent of RA patients developing scleritis, 0.9% in this study compared to 0.7% in previous reports. Only a fraction of patients with scleritis also had a systemic autoimmune diagnosis: 31.4%. This is alignment with the percentage found in prior studies [3, 5–7, 9–13]. However, of all systemic associations, RA only accounted for 6.8% in our series compared to the previous estimation of RA causing 1/3 of scleritis cases [2, 12]. This highlights one of the limitations of a retrospective, large database analysis. There is no way to analyze if all of the patients with scleritis had a systemic work-up, and if an autoimmune condition was identified and the diagnosis code was added to their chart to allow for the diseases to be associated using the SlicerDicer application. In addition, we could not assess if patients had known rheumatologic disease managed by an outside provider. To address this issue, we included rheumatologic diagnoses from the past medical history as well as laboratory components. However, if labs were performed outside and scanned into the system rather incorporated into the EMR, these could have been overlooked. Individual charts were not reviewed to assess if each patient met diagnostic criteria, if the diagnosis was coded accurately, to determine the involvement of one or both eyes, or the anatomic location of the scleritis. As mentioned previously, this study was not executed using a case-control design to assess the frequency of occurrence for each disease or to assess if a patient had multiple rheumatologic or ocular inflammatory diagnoses. Similarly, not all patients with an autoimmune disorder may have had an eye exam or diagnostic codes for scleritis added from a patient’s prior history. Potentially, our search methods may have missed patients followed by outside ophthalmologists or scleritis prior to our search period.

The most interesting findings of the study were the association of Sjogren syndrome and JIA with scleritis in 4.5 and 0.3% of patients, respectively. There have been no prior reported cases of scleritis and JIA, only one case report of peripheral ulcerative keratitis [18]. Similarly, there has been one case of episcleritis associated with JIA [19]. This result reflects on the limitations listed in the previous paragraph with one addition. A subset of JIA patients are HLA-B27 positive, and having HLA-B27 positivity has been independently associated with the development of scleritis [20, 21]. Thus even in pediatric patients, scleritis should be considered in the differential diagnosis, especially in those who are HLA-B27 positive.

This leads to an important point in regards to Sjogren syndrome, which is labelled as primary if another rheumatologic disease is not diagnosed through a laboratory work-up and physical examination. Sjogren syndrome can also be secondary if another unifying diagnosis is made and also included as a diagnosis code, which highlights the inability to discern the difference using ICD-10 and SNOMED terms. This may partially explain the strong association with Sjogren syndrome with scleritis, as previously only case reports and the minority of patients in longitudinal series described the occurrence of scleritis is patients with Sjogren syndrome [22–25].

Given potential pitfalls and limitations with the use large data analyses, future research is needed to delineate associations of systemic disease with the anatomic location and necrotizing subset of scleritis using electronic medical records. However, the results of this study can assist clinicians in broadening their differential diagnoses and work-up during the evaluation of patients with autoimmune conditions and scleritis.

## Data Availability

All data generated or analysed during this study are included in this published article and its supplementary information files.
